# Supplementary material for: Quantification of human enteric viruses as alternative indicators of fecal pollution to evaluate wastewater treatment processes
Source: PeerJ. 2022 Feb 14;10:e12957. doi: 10.7717/peerj.12957 (PMC8852272; doi:10.7717/peerj.12957)
Supplement: Supplemental Information 1 [file peerj-10-12957-s001.docx]

| **Event** | **Sample** | **DNA concentration (ng/µL)** | **RNA concentration (ng/µL)** |
| --- | --- | --- | --- |
| 1 | RS | 5.12 | 21.2 |
|  | AS | 9.08 | 13.9 |
|  | EF | 7.42 | 15.7 |
| 2 | RS | 2.34 | 4.8 |
|  | AS | 2.88 | 13.3 |
|  | EF | 2.36 | 11.3 |
| 3 | RS | 7.58 | 16.3 |
|  | AS | 2.54 | 6.96 |
|  | EF | 6.54 | 10.7 |
|  | SC | 9.16 | 11.9 |
| 4 | RS | 3.14 | 45.4 |
|  | AS | 7.25 | 18.56 |
|  | EF | 9.36 | 13.1 |
|  | SC | 9.72 | 15.9 |
