# Supplementary material for: Quantification of human enteric viruses as alternative indicators of fecal pollution to evaluate wastewater treatment processes
Source: PeerJ. 2022 Feb 14;10:e12957. doi: 10.7717/peerj.12957 (PMC8852272; doi:10.7717/peerj.12957)
Supplement: Supplemental Information 2 [file peerj-10-12957-s002.docx]

| **Package name** | **Version** | **Reference** |
| --- | --- | --- |
| devtools | 2.4.2 | Wickham, Hester, & Chang, 2021 |
| dplyr | 1.0.6 | Wickham, François, Henry, & Müller, 2021 |
| Formula | 1.2-4 | Zeileis & Croissant, 2010 |
| ggplot2 | 3.3.4 | Wickham, 2016 |
| lattice | 0.20-41 | Sarkar, 2008 |
| mvtnorm | 1.1-2 | Genz, et al., 2021 |
| plyr | 1.8.6 | Wickham, 2011 |
| readxl | 1.3.1 | Wickham & Bryan, 2019 |
| report | 0.3.5 | Makowski, Ben-Shachar, Patil, & Lüdecke, 2020 |
| rstudioapi | 0.13 | Ushey, Allaire, Wickham, & Ritchie, 2020 |
| scales | 1.1.1 | Wickham & Seidel, 2020 |
| survival | 3.2-10 | Therneau, 2021 |
| usethis | 2.0.1 | Wickham & Bryan, 2021 |
