# Supplementary material for: Quantification of human enteric viruses as alternative indicators of fecal pollution to evaluate wastewater treatment processes
Source: PeerJ. 2022 Feb 14;10:e12957. doi: 10.7717/peerj.12957 (PMC8852272; doi:10.7717/peerj.12957)
Supplement: Supplemental Information 3 [file peerj-10-12957-s003.docx]

|  | **PC1** | **PC2** | **PC3** | **PC4** | **PC5** | **PC6** | **PC7** | **PC8** | **PC9** | **PC10** | **PC11** | **PC12** |
| --- | --- | --- | --- | --- | --- | --- | --- | --- | --- | --- | --- | --- |
| **Standard deviation** | 3.7772 | 2.8789 | 1.7948 | 0.46159 | 0.07368 | 0.06347 | 0.02625 | 1.645e-14 | 1.462e-16 | 1.156e-16 | 8.057e-17 | 6.175e-17 |
| **Proportion of variance** | 0.5487 | 0.3188 | 0.1239 | 0.00819 | 0.00021 | 0.00015 | 0.00003 | 0.000e+00 | 0.000e+00 | 0.000e+00 | 0.000e+00 | 0.000e+00 |
| **Cumulative proportion** | 0.5487 | 0.8675 | 0.9914 | 0.99961 | 0.99982 | 0.99997 | 1.00000 | 1.000e+00 | 1.000e+00 | 1.000e+00 | 1.000e+00 | 1.000e+00 |
