# Supplementary figures and images for: Quantification of human enteric viruses as alternative indicators of fecal pollution to evaluate wastewater treatment processes
Source: PeerJ. 2022 Feb 14;10:e12957. doi: 10.7717/peerj.12957 (PMC8852272; doi:10.7717/peerj.12957)

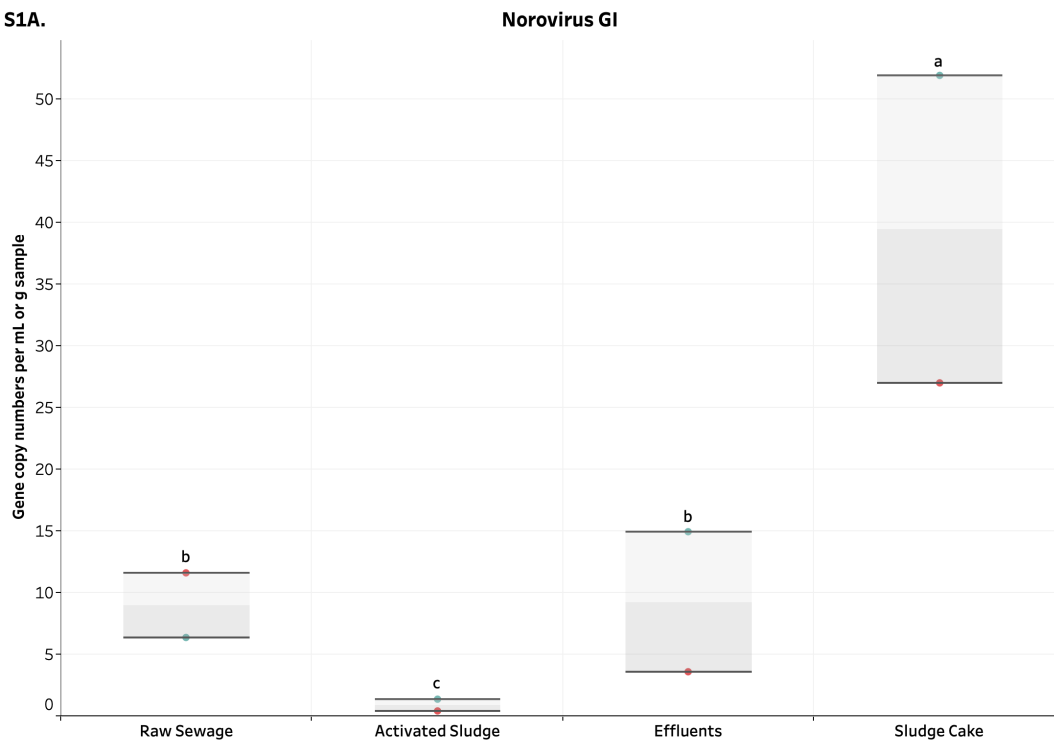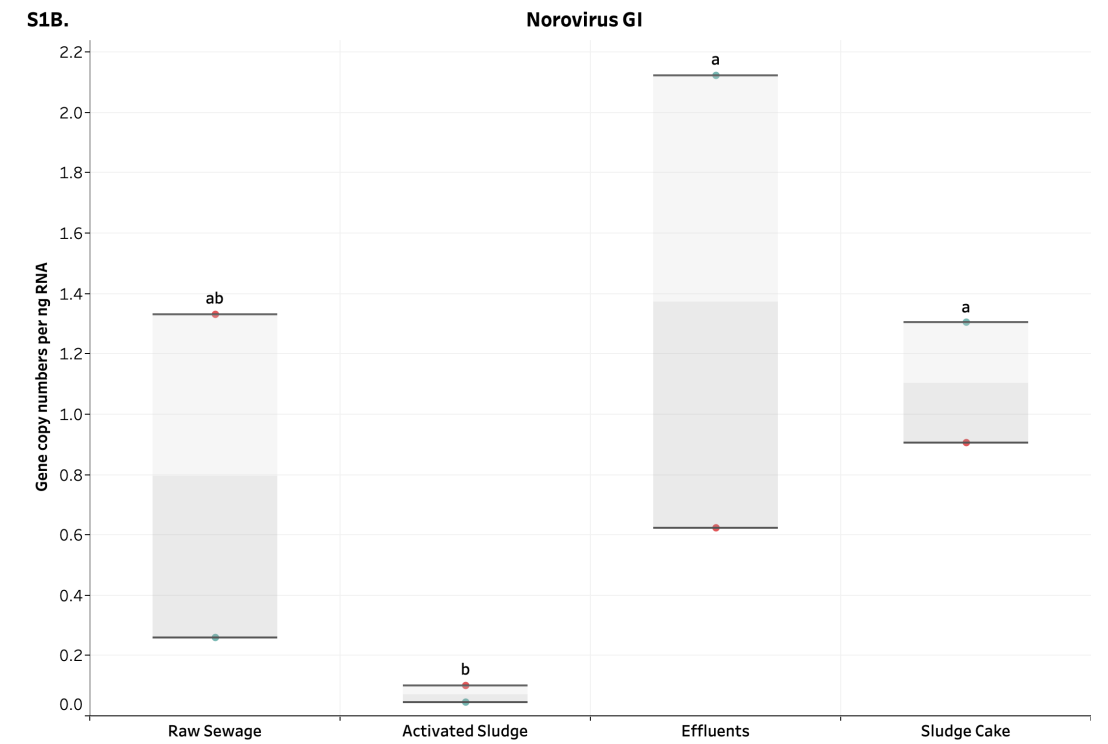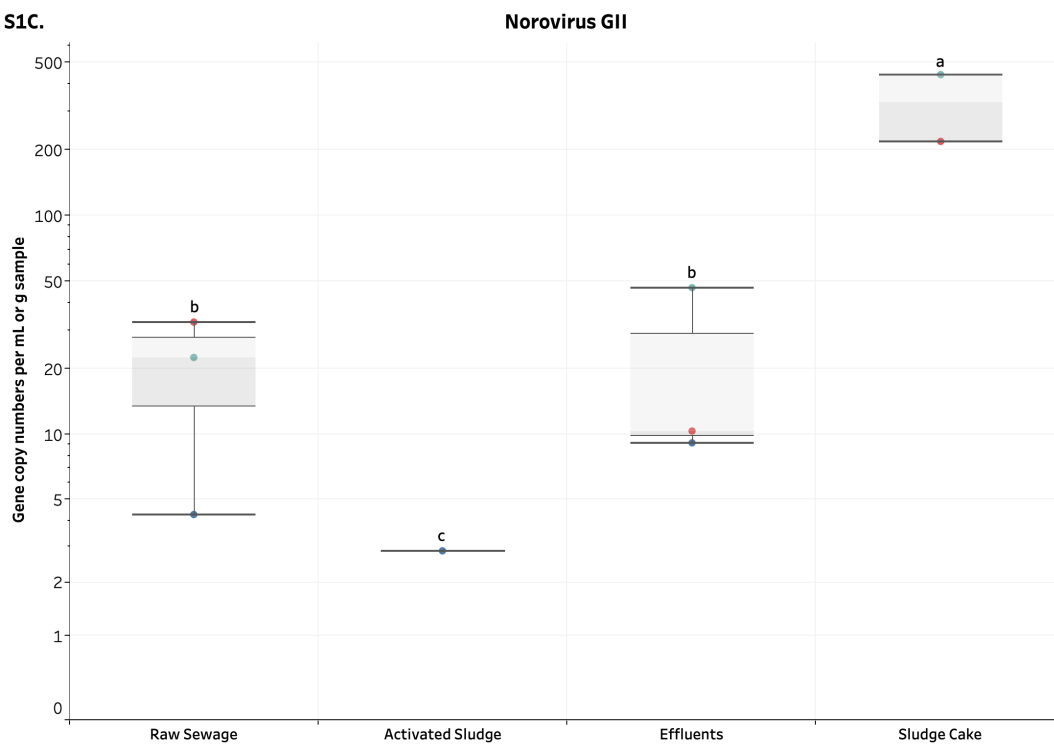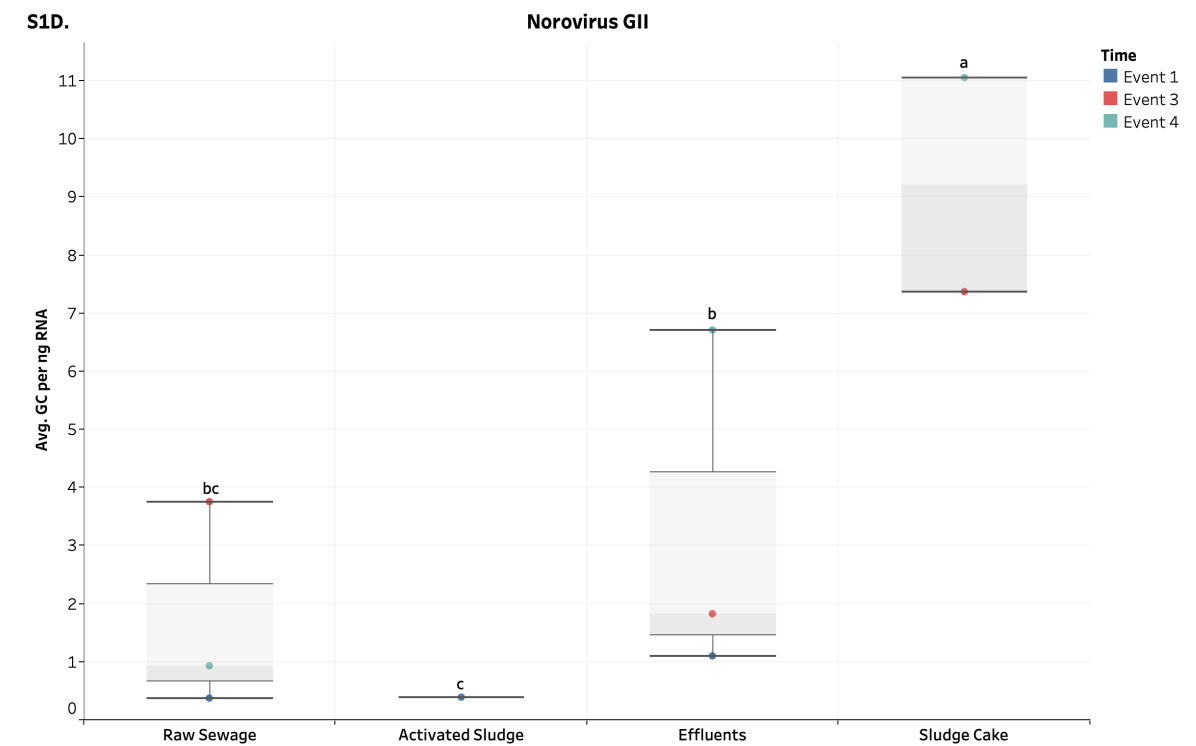

Supplement: Supplemental Information 4 — The unit for the SC in Figs. S1A, S1C is gene copies per g of sample. In S1C this quantity was log10-transformed for aesthetic purposes. Means with different letters indicate significant differences at the 0.05 level across treatments. [file peerj-10-12957-s004.pdf]

**S2A.****Rotavirus**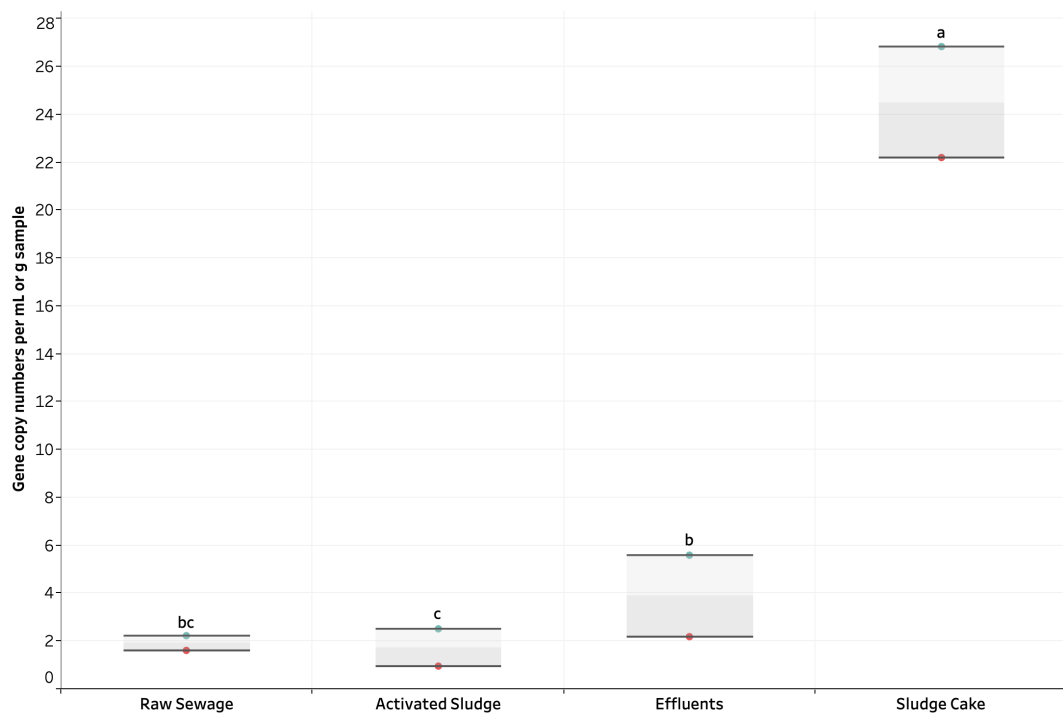**S2B.****Rotavirus**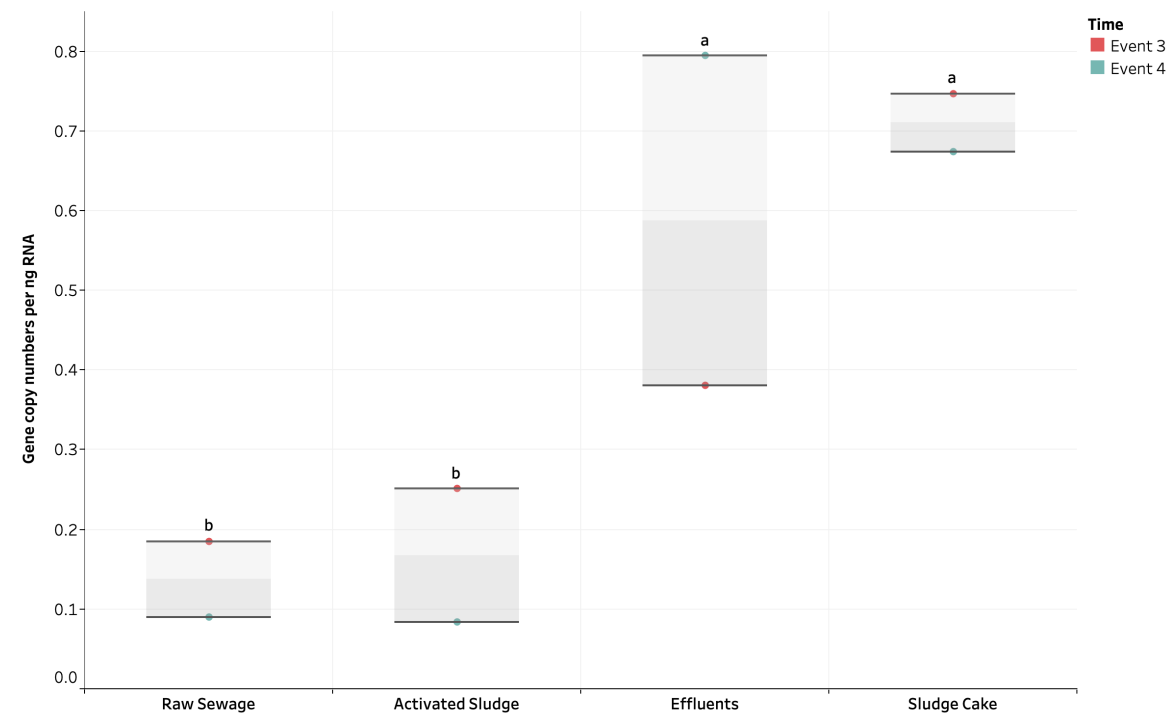

Supplement: Supplemental Information 5 — The unit for the SC in Fig. S2A) is gene copies per g of sample. Means with different letters indicate significant differences at the 0.05 level across treatments. [file peerj-10-12957-s005.pdf]

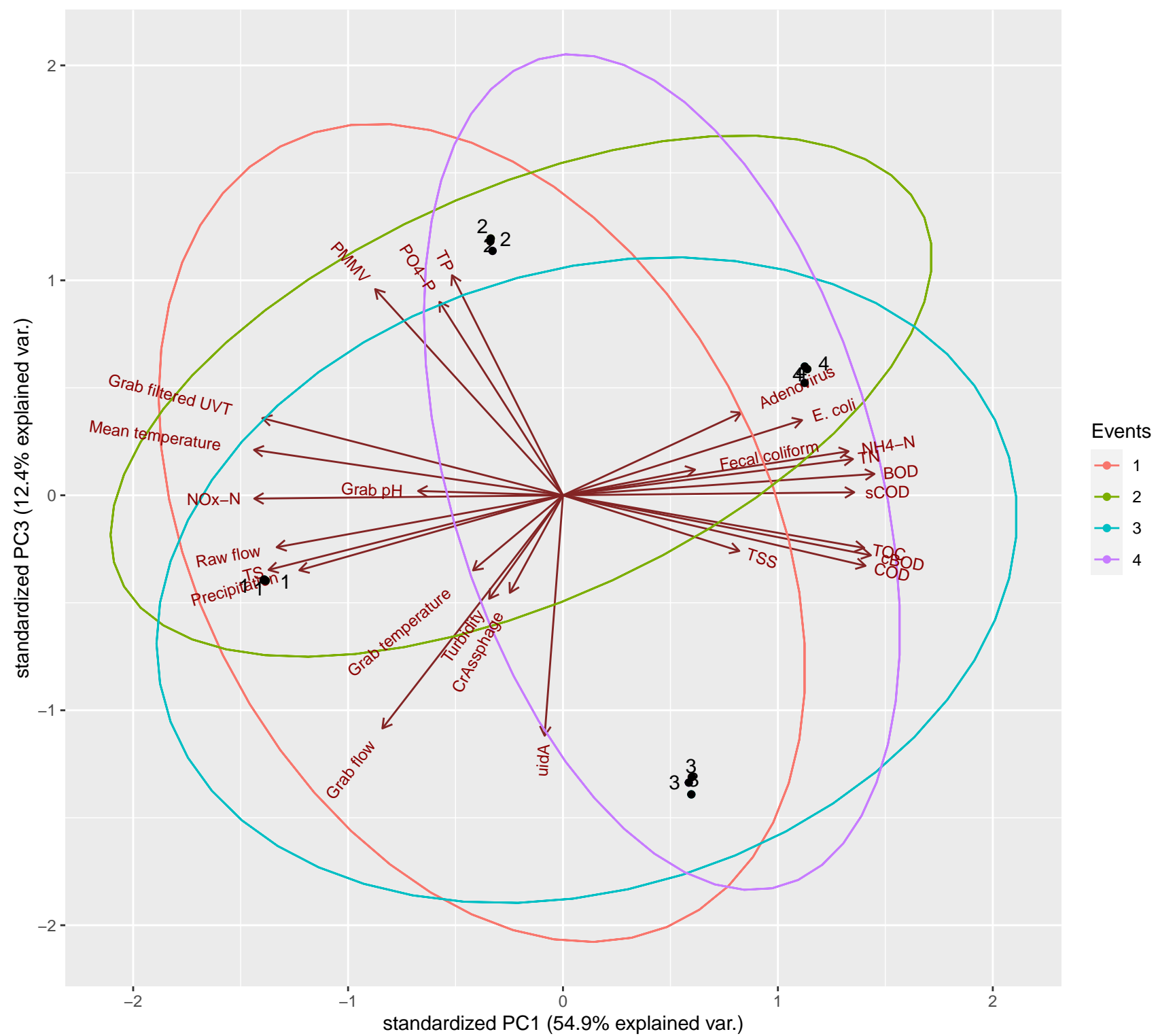

Supplement: Supplemental Information 6 — The only variable not log10-transformed was precipitation due to presence of zero values. [file peerj-10-12957-s006.pdf]

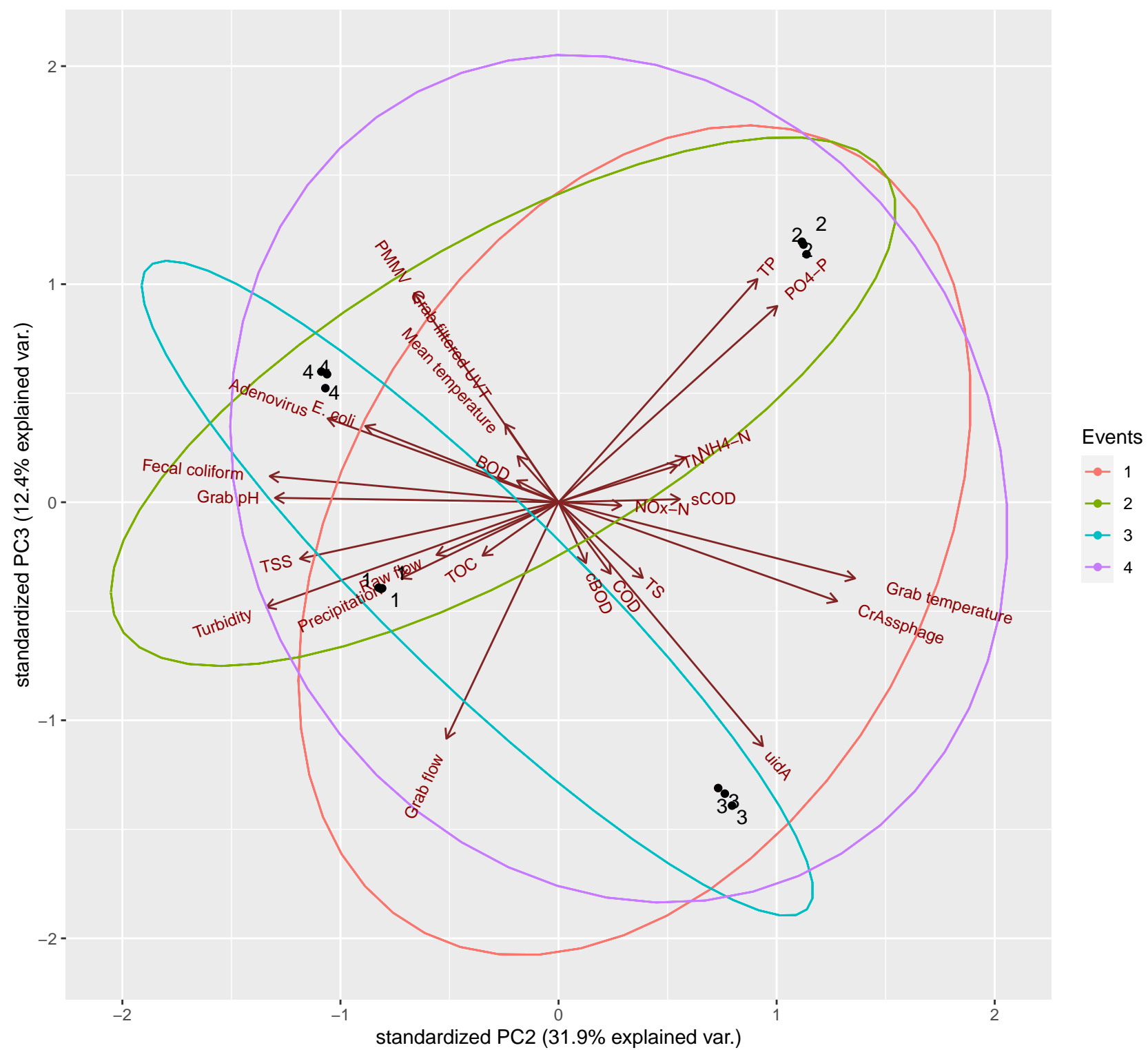

Supplement: Supplemental Information 7 — The only variable not log10-transformed was precipitation due to presence of zero values. [file peerj-10-12957-s007.pdf]
